# The neural milieu of the developing choroid plexus: neural stem cells, neurons and innervation

**DOI:** 10.3389/fnins.2015.00103

**Published:** 2015-03-31

**Authors:** Weerapong Prasongchean, Bertrand Vernay, Zeinab Asgarian, Nahin Jannatul, Patrizia Ferretti

**Affiliations:** ^1^Stem Cells and Regenerative Medicine Section, UCL Institute of Child Health, University College LondonLondon, UK; ^2^Department of Biochemistry and Microbiology, Faculty of Pharmaceutical Sciences, Chulalongkorn UniversityBangkok, Thailand

**Keywords:** chick, choroid plexus, development, innervation, neurogenesis, neural stem cell

## Abstract

The choroid plexus produces cerebrospinal fluid and plays an important role in brain homeostasis both pre and postnatally. *In vitro* studies have suggested that cells from adult choroid plexus have stem/progenitor cell-like properties. Our initial aim was to investigate whether such a cell population is present in vivo during development of the choroid plexus, focusing mainly on the chick choroid plexus. Cells expressing neural markers were indeed present in the choroid plexus of chick and also those of rodent and human embryos, both within their epithelium and mesenchyme. ß3-tubulin-positive cells with neuronal morphology could be detected as early as at E8 in chick choroid plexus and their morphological complexity increased with development. Whole mount immunochemistry demonstrated the presence of neurons throughout choroid plexus development and they appeared to be mainly catecholaminergic, as indicated by tyrosine-hydroxylase reactivity. The presence of cells co-labeling for BrdU and the neuroblast marker, doublecortin, in organotypic choroid plexus cultures supported the hypothesis that neurogenesis can occur from neural precursors within the developing choroid plexus. Furthermore, we found that extrinsic innervation is present in the developing choroid plexus, unlike previously suggested. Altogether, our data are consistent with the presence of neural progenitors within the choroid plexus, suggest that at least some of the choroid plexus neurons are born locally, and show for the first time that choroid plexus innervation occurs prenatally. Hence, we propose the existence of a complex neural regulatory network within the developing choroid plexus that may play a crucial role in modulating its function during development as well as throughout life.

## Introduction

The choroid plexus (CP) is important for the secretion of cerebrospinal fluid (CSF) and plays an important role in brain development, homeostasis and disease. The CP, found in lateral, third and fourth ventricles of the brain, consists of a highly vascularized stroma with loose connective tissue surrounded by a single layer of simple cuboidal epithelium. The CP epithelium, considered to be a specialized ependymal epithelium, is continuous with the ependyma lining the brain of lateral, III and IV ventricles. It originates from the neuroepithelium, whereas the inner CP stromal core is believed to originate from the mesenchyme (Lehtinen et al., 2013; Liddelow et al., 2013).

The CP develops early during embryogenesis and which CP is first clearly visible depends on the species. In mouse and humans, the IV ventricle CP anlage is the first one observed at approximately E10.5 and 41 days of gestation, respectively (Dziegielewska et al., 2001; Hunter and Dymecki, 2007). In the chick embryo, it has been indicated that a CP anlage is first detectable in the lateral ventricles between E6 and E8. The chick lateral ventricle CP rapidly grows, lengthening and branching out many times with a dip in its growth reported at around E15–E16 followed by its thinning and flattening (Smith, 1966; Stastny and Rychter, 1976).

The CP constitutes the blood-CSF barrier that is established by the tight junctions of the CP epithelial cells. The CP is a very active organ that continuously secretes CSF from early development throughout life (Johansson et al., 2008; Liddelow et al., 2012; Lehtinen et al., 2013). It has been proposed that its secretory function is controlled by its autonomic innervation (Edvinsson and Lindvall, 1978; Lindvall and Owman, 1981). CSF secretion could be inhibited by stimulation of sympathetic nerves in the neck (Lindvall et al., 1978c) and via activation of β2 receptors (Nathanson, 1980). Sympathetic denervation in rabbits resulted in hydrocephalus and was fatal (Lindvall and Owman, 1981). It has been assumed that nerves enter the CP only after birth (Tsuker, 1947; Edvinsson et al., 1973; Lindvall et al., 1977a, 1978a,b,c; Edvinsson and Lindvall, 1978), but there is only one study where this issue was specifically addressed and that suggests that the CP becomes innervated postnatally (Lindvall and Owman, 1978). This seems peculiar, as innervation tends to occur at early stages of organ development, before a high degree of complexity is achieved. We therefore postulated that this would be the case also for the CP and set to test this hypothesis.

A variety of stem/progenitor cells have been hypothesized to reside within the CP. Nataf and co-workers have reported that myeloid progenitors in the CP are capable of differentiation toward macrophage or dendritic cell phenotypes (Nataf et al., 2006). In addition, the CP has been reported to contain neural stem-like cells able to give rise to neural cells *in vitro* and upon transplantation into a spinal cord injury model (Kitada et al., 2001). The transcriptome of the CP from adult mouse has revealed the presence of genes important for neural development (Itokazu et al., 2006; Marques et al., 2011). The neural stem cell potential observed in the CP has been suggested to reside in the CP epithelium on the basis of *in vitro* studies (Itokazu et al., 2006). The localization of those putative neural stem cell population(s) was not extensively studied *in vivo*. In another study in rat pups, the presence of nestin-expressing cells just beneath the CP epithelium, that decreased in number with age, was taken to indicate the presence of neural stem cells in the CP stroma (Huang et al., 2011). We hypothesized that the developing CP harbors a neurogenic niche that may account for the proposed “neurogenicity” of the CP.

We assessed the presence of neural progenitors and time of innervation in developing CPs, focusing on the chick CP *in vivo* and in organotypic cultures. We show for the first time that the CP mesenchymal compartment contains neural progenitor-like cells that may be the precursors of the neurons identified in this compartment, and that innervation of the CP is an early developmental event. Hence, we suggest a model where a neural regulatory network is present within the CP and may play a crucial role in modulating its function during development as well as throughout life.

## Materials and methods

Unless otherwise specified all general reagents were from Sigma and tissue culture reagents from Gibco.

### Chicken embryos and isolation of choroid plexus (CP)

Fertilized Brown Leghorn chicken eggs were purchased from Henry Stewart & Co. Ltd (Lincolnshire, UK). On arrival, eggs were stored at 15°C in the egg fridge (JENCONS Ltd., USA) and used within 1 week. They were maintained on cardboard egg racks in a humidified forced flow incubator at 38°C (MARSH automatic incubator, LYON electric company, USA) until the required developmental stages. Chick embryos were decapitated and brains removed. The meninges were carefully peeled off because its connective tissue normally attaches to the pineal gland which also connects to the 3rd ventricle CP. The isolated CP was used for whole mount immunohistochemistry, organotypic culture, or RNA extraction. Unless otherwise indicated, lateral ventricle CPs were used.

### Human and mouse embryos

Mouse embryonic brains were isolated and fixed in 4% paraformaldehyde (PFA) overnight at 4°C, cryo-protected by incubation in 30% sucrose containing 0.02% sodium azide in PBS (phosphate buffered saline) at 4°C for approximately 24 h, OCT embedded, and immunohistochemistry performed. Paraffin sections of human brains at Carnegie stage 23 (Cs23; 56 days of gestation) were obtained from the Human Developmental Biology Resource (HDBR).

### Immunohistochemistry and whole mount labeling

Embryonic chick or mouse brains were fixed in 4% PFA overnight at 4°C, cryo-protected by incubation in 30% sucrose containing 0.02% sodium azide in PBS (phosphate buffered saline) at 4°C for approximately 24 h, OCT embedded and cryosectioned (14–16 μm thick). Isolated CPs were fixed in 4% PFA overnight at 4°C and washed several times with PBS for whole mount labeling. For wax removal and epitope unmasking, human embryonic brain sections were immersed in 1:20 Declere® solution (Sigma) in PBS, heated in a microwave oven at 720 watt for 5 min and then at 270 watt for 20 min, rinsed in PBS and then processed as cryosections. Brain sections or whole mount CPs were permeabilized (0.5% Triton X-100 in PBS) and blocked in 20% BSA in PBS for 1 h at room temperature. Sections were incubated with primary antibodies (Table 1) diluted in blocking solution for 3–4 h at room temperature or overnight at 4°C. After several washes with PBS, sections were incubated with appropriate secondary antibodies for 1–2 h. Citiflour® solution (Citifluor Limited) was used as a mounting medium in most of immunohistochemistry for cryosections and paraffin sections. In this case, cell nuclei were visualized with Hoechst 33258 (2.5 μg/ml final concentration). Stained section and whole mount CPs were visualized and digitally scanned using an Axiophot 2 (Zeiss) with Hamamatsu ORCA-ER digital camera or by confocal laser scanning microscopy using an LSM 710 (Zeiss). Image analysis was performed using ImageJ software (Rasband, 1997-2009).

**Table 1:** **List of antibodies used for immunohistochemistry and whole mount staining**.

	**Dilution**	**Host**	**Types**	**Suppliers**
ANTIGEN (PRIMARY ANTIBODIES)^*^				
Aquaporin 1 (Aq-1)	1: 200	Mouse	IgG	Bioscience
βTub3	1: 1000	Mouse	mAb IgG	Promega,
BrdU	1: 100	Mouse	IgG	DSHB
Clusterin	1: 100	Rabbit	pAb IgG	Santa cruz (SC)
Doublecortin (Dcx)	1: 100	Goat	IgG	Cell signaling
Flk-1	1: 200	Rabbit	pAb, IgG	Fisher scientific
Glial fibrillary acidic protein (GFAP)	1: 1000	Rabbit	pAb	Chemicon
Laminin	1: 200	Rabbit	pAb	Milipore
Myelin basic protein	1: 100	Mouse	IgG	Chemicon
Nestin (human)	1: 1000	Rabbit	pAb IgG	Milipore
Nestin (rat, mouse)	1: 200	Mouse	IgG	Developmental studies hybridoma bank (DSHB)
NF200	1: 100	Rabbit	IgG	Sigma
Otx2	1: 200	Rabbit	pAb IgG	Abcam
P75NTR	1: 500	Rabbit	IgG	Sigma
Pax6	1: 100	Mouse	mAb IgG1	DSHB
Phosphohistone3 (pH3)	1: 100	Rabbit	IgG	Upstate
Sox2	1: 1000	Rabbit	pAb	Milipore
Synaptic vesicles 2 (SV2)	1: 100	Mouse	IgG1	DSHB
Transitin (chick)	1: 100	Mouse	mAb IgG1	DSHB
Tyrosine hydroxylase	1: 400	Sheep	pAb	Millipore
VEGF	1: 100	Mouse	mAb IgG1	SC
Vimentin	1: 100	Mouse	IgG	DAKO
ZO-1	1: 100	Rabbit	pAb IgG	Molecular Probe
**SECONDARY ANTIBODIES**				
Anti-mouse IgG1a Alexa Fluor® 568	1: 400	Goat	–	Molecular probe
Goat anti-mouse IgM Alexa Fluor® 594	1: 400	Goat	–	Molecular probe
Anti-rabbit Alexa Fluor® 594	1: 400	Goat	–	Molecular probe
Anti-mouse IgG Alexa Fluor® 488	1: 400	Goat	–	Molecular probe
Anti-sheep Alexa Fluor® 488	1: 1000	Rabbit	–	Molecular probe

*mAb, monoclonal antibody; pAb, polyclonal antibody*.

* *The same antibodies were used in the different species unless the species is indicated in brackets*.

### Organotypic culture of chick CPs

E12 chick embryo CPs were used to set up organotypic cultures. CPs from lateral and third ventricles were dissected and grown onto a 0.4-μM-Millipore®-CM culture plate insert in MEM (minimal essential medium) containing Glutamax, 5% Earle's balanced salt solution (EBSS), 36 mM D-glucose, 1% penicillin/streptomycin and 25% horse serum.

### MTT assay and PI staining of organotypic chick CP cultures

Propidium iodide (PI) was used to identify cell death in organotypic CP cultures. Briefly, the cultures were incubated with culture medium containing 5 μg/ml PI for 1 h at 37°C in an atmosphere of 95% O_2_ and 5% CO_2_. Organotypic cultures were incubated with 0.5 mg/ml MTT [3-(4,5-Dimethylthiazol-2-yl)-2,5-diphenyltetrazolium bromide] solution for 1 h at 37°C to assess cell viability. The appearance of blue/purple formazan crystals is indicative of cell viability and structural integrity.

### BrdU incorporation in organotypic chick CP cultures

Organotypic CP preparations were treated with 10 μM BrdU for 3 days and then processed for BrdU detection and immunohistochemistry. CPs were incubated in 1N HCl for 10 min on ice, followed by incubation in 2N HCl for 10 min at room temperature, and then for 20 min at 37°C. The CPs were then washed three times in 0.1M borate buffer at room temperature, once in PBS, and immunostained as described above.

### Polymerase chain reaction (RT-qPCR)

RNA was extracted from chick lateral ventricle CP and midbrain at different developmental stages (see Results) using Tri-Reagent (Life Technologies) according to the manufacturer's instructions. RNA was retro-transcribed with Moloney murine leukemia virus reverse transciptase (Promega, Madison, WI). The annealing temperature for RT-PCR was 54°C for Sox2 and Otx2 (30 cycles) and 66°C for GAPDH (25 cycles). Real time quantitative polymerase chain reaction (RT-qPCR) was preformed with ABI Prism 7500 sequence detection system (Applied Biosystems) using the QuantiTect SYBR Green PCR kit (Qiagen, Hilden, Germany) according to the manufacturer's instructions. Gene expression data were normalized using GAPDH housekeeping gene as a reference using the 2-ΔΔCt method. The primers used for RT-PCR and RT-qPCR are listed in Table 2.

**Table 2:** **Primers used for analysis of gene expression in chick choroid plexus and midbrain by RT-PCR and RT-qPCR**.

**Gene**	**Primers (5′-3′)**
GAPDH	For CCAGGTTGTCTCCTGTGACT Rev CACAACACGGTTGCTGTAT
Transitin	For CTGGAGCAGGAGAAGCAGAG Rev CTGTTGGCCAGCTTGAACTC
GFAP	For CCAGGTTGTCTCCTGTGACT Rev CACAACACGGTTGCTGTAT
Doublecortin (Dcx)	For CCCATTCGTTTGAGCAAG TT Rev CCTGTGCATAGCGGAATTTT
ß3-tubulin	For TCTCACAAGTACGTGCCTCG Rev CCCCGCTCTGACCGAAAATG
Sox2	For AGGCTATGGGATGATGCAAG Rev GTAGGTAGGCGATCCGTTCA
Otx2	For CCACCTCAACCAGTCTCCAG Rev TTCCATGAGGATGTCTGGTC

### Statistical analysis

Data are presented as mean ±SEM. The statistical analysis was performed using GraphPad Prism version 5.00 for Windows. Statistical significance was evaluated by One-Way ANOVA. A *p*-value equal to or less than 0.05 was considered as statistically significant.

## Results

### Expression of neural markers in the developing choroid plexus (CP)

**Figure 1:**
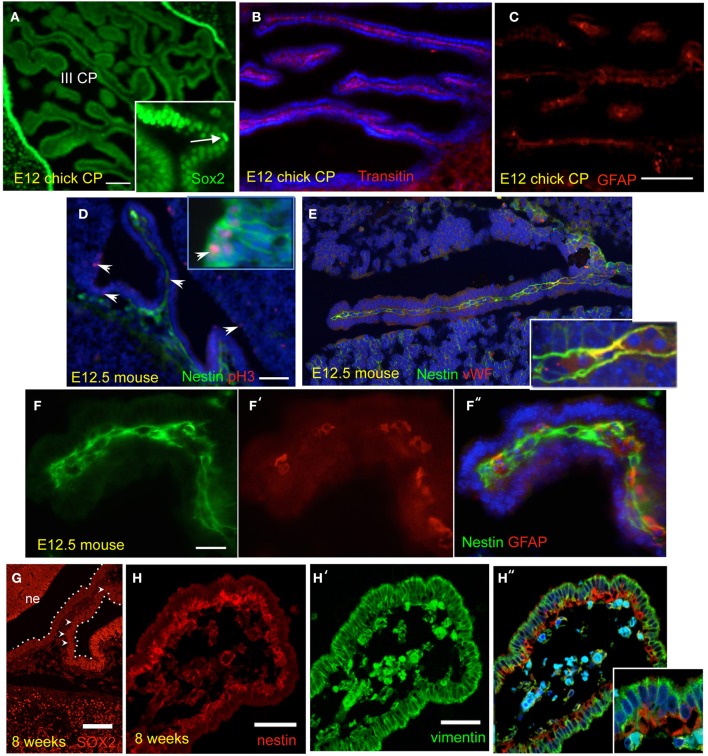
**Neural progenitor markers are expressed in the developing choroid plexus (CP), in different species**. Unless otherwise indicated, micrographs show the CP of lateral ventricles. **(A–C)** E12 Chick CP stained for Sox2, transitin, and GFAP. III CP: third ventricle CP. **(D–F)** E12.5 mouse CP stained for pH3 (phosphorylated-histone 3), nestin, von Willebrand factor, and GFAP, either alone or in combination. Arrowheads in **(D)** point at proliferating cells in the CP and in the neuropepithelium (insert); only the CP is shown in **(F)**. **(G,H)** Human CP at 8 weeks of gestation (CS23) stained for Sox2, nestin, and vimentin. The CP is outlined and some of the brighter SOX2-positve cells in the CP are indicated by arrowheads; note the gradient of SOX2 staining in the CP; ne, neuroepithelium. Nuclei are counterstained with Hoechst dye (blue). Scale bars are: **(A)** = 50 μm; **(B,C)** (same magnification) = 100 μm; **(D,E)** (same magnification) = 100 μm; **(F)** = 100 μm; **(G)** = 100 μm; **(H)** = 50 μm.

**Figure 2:**
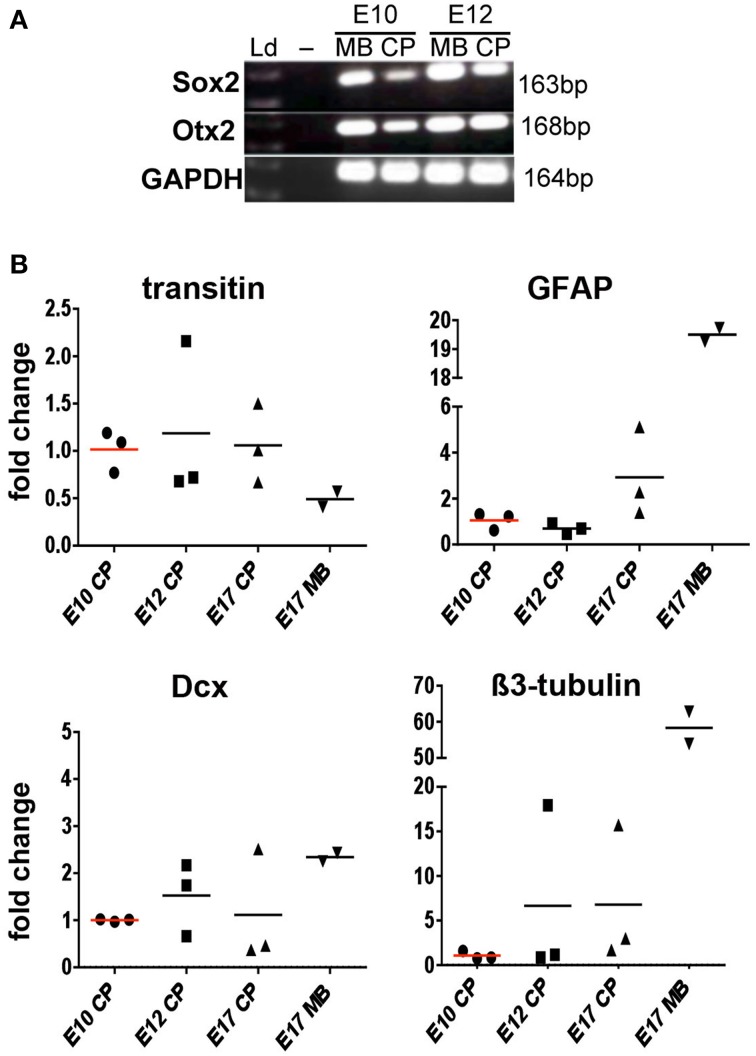
**Expression of neural transcripts in the chick lateral ventricle choroid plexus (CP) at E10, E12, and E17 detected by RT-PCR and RT-qPCR. (A)** Detection of Sox2 and Otx2 by RT-PCR in the CP and midbrain (MB, positive control). **(B)** Detection of transitin, GFAP, Dcx, and β3-tubulin by RT-qPCR. Fold changes in individual CPs or MB normalized against GAPDH are shown. Note that transitin, GFAP, Dcx, and β3-tubulin transcripts are detected in the CP at all developmental stages examined; no statistically significant differences in expression levels are observed.

Expression of markers of neural stem/progenitor cells, such as Sox2 and nestin, in the CP was first studied by fluorescent immunohistochemistry in sections from E12 chick brains (Figure 1 and Table 3). In the E12 CP, cells positive for the neural progenitor marker, Sox2, were detected in the CP epithelium of both lateral and 3rd ventricles (Figure 1A). The staining intensity in the CP was lower than in the neuroepithelium and a gradient of Sox2 expression, from high, in the neuroepithelium, to low, in the CP epithelium, was apparent (Figure 1A, Table 3). Interestingly, Sox2 expression in cells located at presumed CP branching points was stronger than in adjacent cells (see insert Figure 1A). The early CP epithelium was also positive for Otx2 and Pax6, and for several other markers that have been reported to be expressed in neural stem cells (Table 3). In contrast to the CP epithelium, the CP stroma was Sox2-negative. However, the CP mesenchyme contained cell expressing other neural markers, transitin, the chick nestin-like protein, and GFAP, a marker of astrocytes, radial glia and neural stem cells (Figures 1B,C). Expression of neural progenitor-associated proteins in the chick CP was consistent with expression of their transcripts detected by RT-PCR (Figure 2).

**Table 3:** **Summary of protein expression in the developing chick choroid plexus**.

	**Stroma**		**Epithelium**	
	**E12**	**E19/20**	**E12**	**E19/20**
**NEURAL STEM/PROGENITOR CELL**				
Sox2	–	nd	✓	nd
Otx2	–	–	✓	✓
Vimentin^*^	✓ (low)	✓ (low)	✓ (high)	✓ (high)
Transitin	✓	√	–	–
GFAP	✓	✓	–	–
Cytokeratin	-	nd	✓	nd
Doublecortin	✓	✓	–	–
**NEURONAL**				
ß3-tubulin	✓	✓	–	–
Neurofilament	✓	✓	–	–
Synaptic vesicles (SV2)	✓	✓	–	–
Tyrosine hydroxylase	✓	nd	–	nd
**TIGHT JUNCTION PROTEINS**				
ZO-1	–	–	✓	✓
ASPP2	✓	✓	✓	✓
Connexin-34	–	–	✓	✓
**OTHERS**				
Laminin	✓	nd	–	nd
Aquaporin-1	–	nd	✓	nd
Clusterin^*^	✓ (low)	✓ (low)	✓ (high)	✓ (high)

–, negative; nd, not done;

* *particularly low levels of expression in the CP stroma and high levels of expression in the CP epithelium are indicated*.

In order to establish whether neural progenitor-like cells were present only in the chick or also in mammals, we studied their expression in mouse CP at embryonic day 12.5 (E12.5) and in the Cs23 (56 days of gestation) human CP. Nestin and GFAP protein expression was found in the stromal compartment of the mouse CP paralleling that in the chick (Figures 1D,E). Proliferating nestin-positive cells were observed in the CP as well as in the neuroepithelium (Figure 1D). Expression of nestin and GFAP appeared to be mutually exclusive (Figure 1F), but some overlap of nestin immunoreactivity with an endothelial marker, the von Willebrand factor, was observed suggesting expression of nestin in some endothelial cells (Figure 1E). The developing human CP at Cs23 also displayed a gradient of SOX2 staining, as observed in the chick, and was also positive for vimentin and nestin (Figures 1G,H). Nestin staining in the human CP appeared to be sub-epithelial and vimentin expression was very high in the CP epithelium but not restricted to it (Figure 1H). This paralleled the vimentin pattern of expression observed in the chick CP (Table 3). In mouse CP at comparable stages of development, vimentin was detected in the CP stroma (not shown).

### Analysis of neurogenesis in the developing chick choroid plexus (CP)

Given the expression of several neural markers observed in the CP, we investigated whether neurons were generated within the CP focusing on the chick CP.

**Figure 3:**
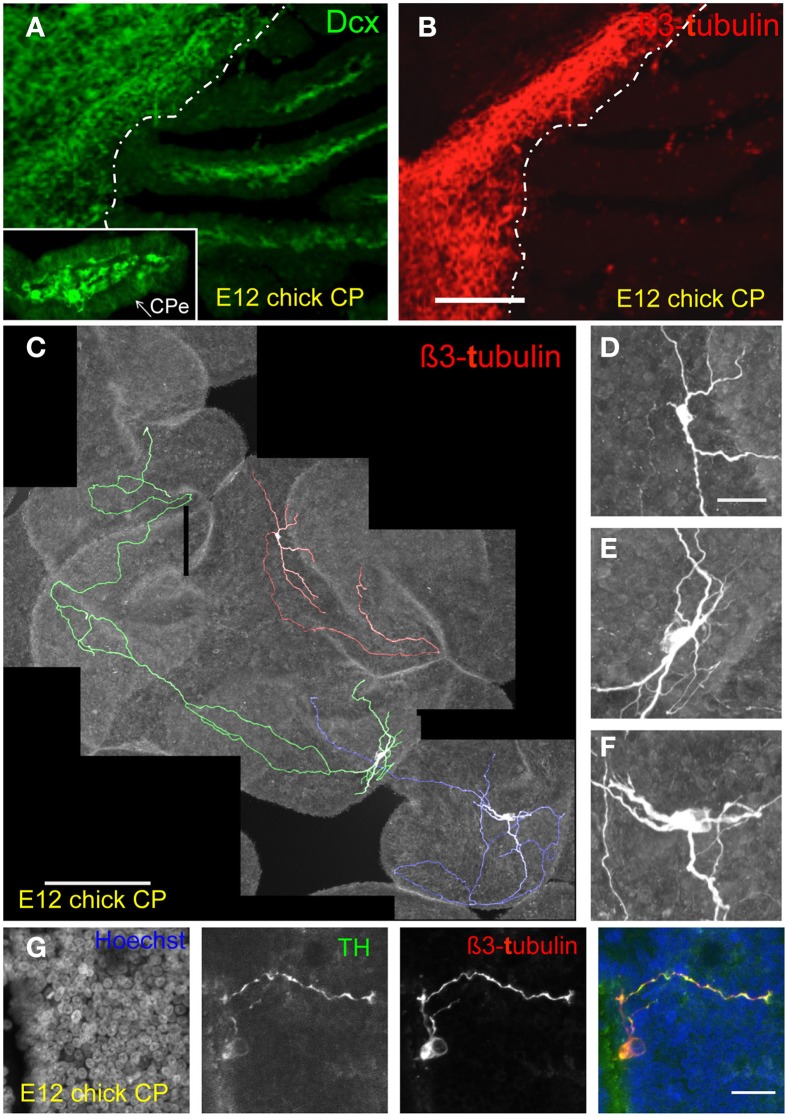
**Identification of neuroblasts and neurons in the developing chick CP. (A,B)** E12 lateral CP section stained for doublecortin (Dcx) and ß3-tubulin in E12 chick CP. Note Dcx reactivity in the CP stroma (insert in A) as well as in the brain; only some punctuated staining is observed in the CP with the anti-ß3-tubulin antibody whereas the brain tissue is strongly labeled. The approximate boundary between brain and CP is indicated by the dotted line. CPe, CP epithelium. **(C–F)** Examples of extensive branching (individual neurons in **(C)** are shown in different colors for ease of visualization) and neuronal morphologies detected by ß3-tubulin staining. **(G)** Staining of the E12 CP for tyrosine-hydroxylase (TH), ß3-tubulin and Hoechst dye (nuclei) and merged image; note the presence of a positive TH neuron in proximity of the CP epithelium. Scale bars: **(A,B)** (same magnification) = 200 μm; **(C**) = 200 μm; **(D–F)** (same magnification) = 30 μm; **(G)** = 20 μm.

E12 chick lateral ventricle CPs were immunostained for the neuroblast marker, doublecourtin (Dcx), and the neuronal marker, β3-tubulin. Extensive Dcx reactivity was detected even at a low magnification within the CP mesenchyme. Some scattered β3-tubulin reactivity was observed in the E12 CP sections (Figures 3A,B). Both *Dcx* and *β3-tubulin* transcripts were detected in the CP (Figure 2). The presence of cells double-labeled for Dcx and β3-tubulin was also consistent with birth of neurons within the CP (Supplementary Figure 1). As it was difficult to visualize what the anti-β3-tubulin antibody staining in CP sections, particularly at later stages of development when the CPs are highly branched, most of the subsequent analysis was carried out in whole mount CP preparations. Analysis of whole mount E12 CPs stained for β3-tubulin clearly showed the presence of β3-tubulin-positive cells with extensive branching and a variety of neuronal morphologies (Figures 3 C–F, Movie 1).

**Figure 4:**
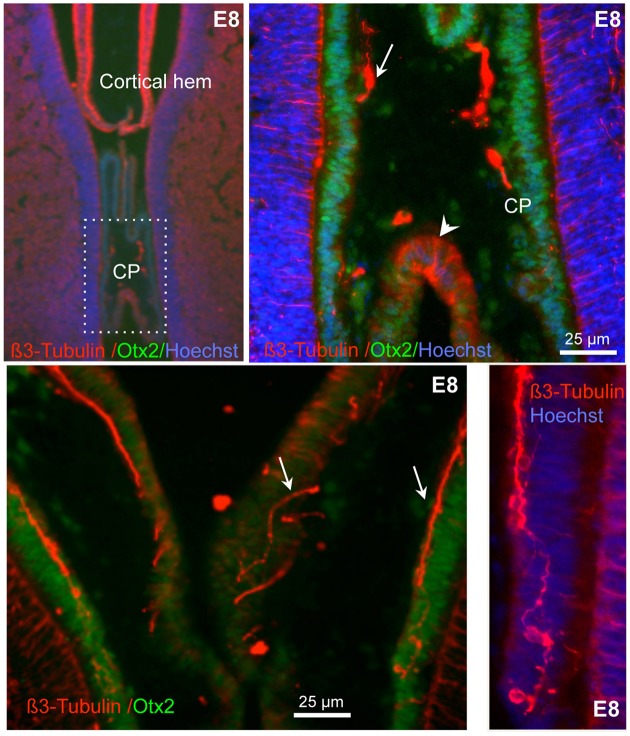
**Expression of β3-tubulin in E8 chick CP**. Sections double-labeled for ß3-tubulin and Otx2, a marker of the CP epithelium. High magnification images are shown in the right panels. Note the presence of nerves and neurons (some indicated by arrows) in the CP as well as of cells spanning across the CP epithelium (arrowhead). Nuclei are counterstained with Hoechst (blue). The bottom right panel is at the same magnification as the one at its left.

**Figure 5:**
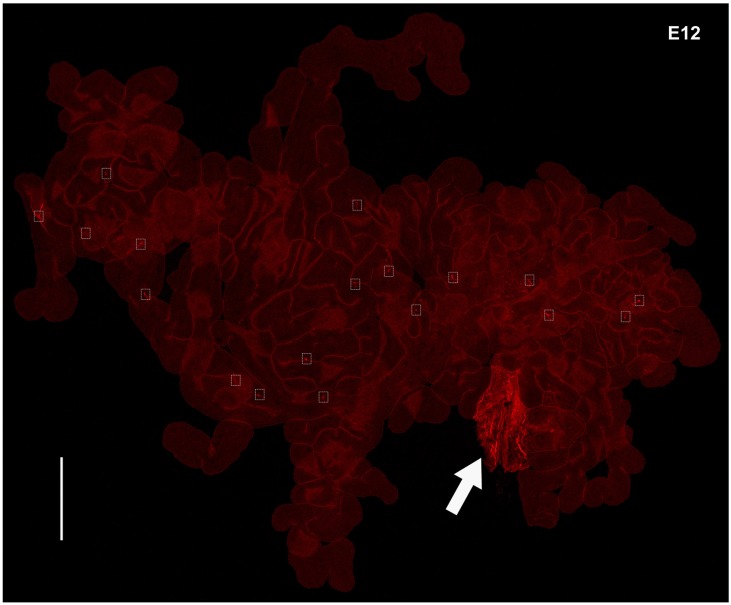
**Expression of β3-tubulin in whole mount E12 chick CP**. The nerve bundle in the CP stalk is indicated by the arrow and some neurons within the CP are boxed. Scale bars = 500 μm.

**Figure 6:**
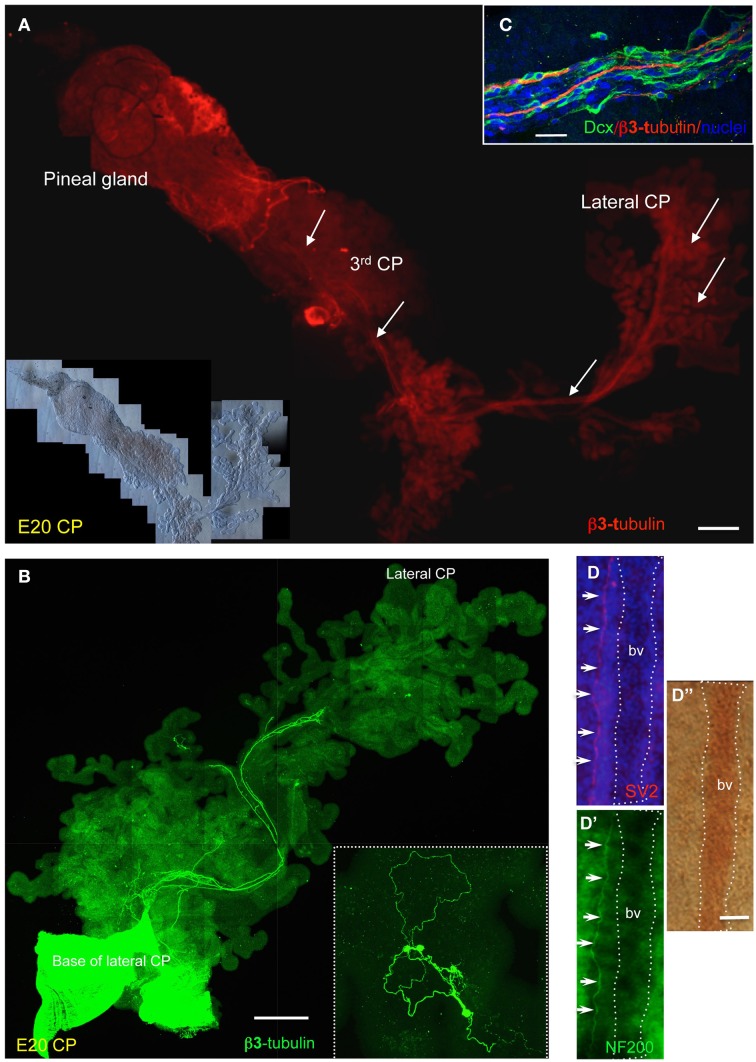
**Expression of neuronal proteins in chick CPs. (A,B)** Examples of β3-tubulin staining in whole mount E20 CPs; note the extensive network connecting lateral ventricle CP, 3rd ventricle CP and pineal gland. The arrows point at some of the neurons observed at high magnification. **(C)** E19 CP stained for doublecortin (Dcx) and ß3-tubulin; nuclei are counterstained with Hoechst dye. **(D–D”)** Whole mount E19 chick CP double-labeled for the synaptic vesicle 2 marker, SV2, and NF200 and corresponding bright field image; nuclei are counterstained with Hoechst dye. The dotted line indicates a blood vessel (bv). Scale bars: **(A)** = 200 μm; **(B)** = 500 μm; **(C,D)** = 25 μm.

We then wished to establish when these neurons first appeared in the CP and whether they were a transient or a stable feature of the CP. Hence we stained chick CPs for β3-tubulin at E6, E8, E10, E18, E19, and E20 (Figures 4–**6**, Supplementary Figure 1). We were unable to identify the CP at E6, but at E8 a small CP was clearly visible, and it was found to contain neurons as indicated by β3-tubulin staining and morphological appearance (Figure 4). At this stage β3-tubulin staining was observed also in some cells spanning the CP epithelium and contacting the CSF; some thin β3-tubulin fibers that appeared to project into the CSF were also observed at E8 as well as at later stages when double-labeling for ß3-tubulin and the tight junction marker, ZO-1, was carried out (Figure 4, Movie 2). Furthermore, nerve fibers that appeared to be entering the CP mesenchyme were observed at E8 as well as at E12 (Figures 4, 5).

The morphology of the CP neurons became more complex with development. At E8 neurons were mainly bipolar, at E10 some were bipolar, some tripolar and other had several branches, and from E12 the neuronal processes appeared to be longer and more convoluted (Figures 3C–F, 4, Movie 1). Staining for tyrosine-hydroxylase at E12 indicated that at least some of the neurons present in the developing CPs are catecholaminergic (Figure 3G, Supplementary Figure 2A). The morphology of some of these neurons resembles the drawing by Clark (1928) shown in Supplementary Figure 2B, though this author interpreted the bulbous structures as nerve terminals.

Nerves coursing the length of the CPs were detected at all stages studied and in all CPs, with dense clusters of neurons or fibers located at the base of the lateral ventricle CP and at branching points, where groups of neurons with highly convoluted morphology could be observed (Figures 4, 5, 6A,B). The CP neural fibers did not appear to be myelinated, as staining for myelin basic protein was only detected in the nerve bundle at the base of the CP stalk (Supplementary Figure 3). Dcx-positive cells were still present at late developmental stages, consistent with detection of *Dcx* mRNA by RT-qPCR (Figure 6C). A high density of nerve terminals/fibers was observed in close proximity to blood vessels, as indicated by staining with the SV2 (synaptic vesicle protein 2) antibody that detects a glycoprotein important for synaptic vesicle function, and NF200; close correspondence between SV2 and NF200 staining could be observed, notwithstanding some background with the NF200 antibody in whole mount CPs (Figure 6D) (Xu and Bajjalieh, 2001).

Whole mount analysis of E20 pineal gland, 3rd and lateral ventricle CPs isolated together revealed an interesting neural network between these structures (Figure 6B). Interconnecting nerve fibers were clearly observed between the pineal gland and the 3rdV CP and between the 3rd and lateral ventricle CPs.

### Analysis of choroid plexus (CP) organotypic cultures

**Figure 7:**
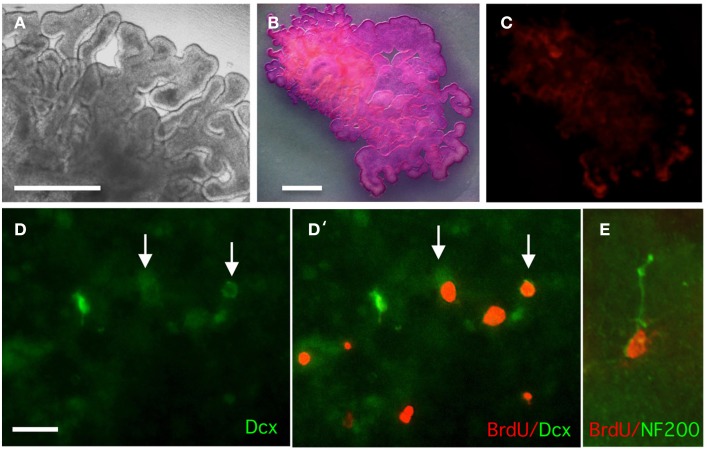
**Analysis of survival, proliferation, and doublecortin (Dcx) expression in organotypic cultures of E12 lateral CP. (A)** Phase image of CP after 7 days in culture. **(B)** MTT staining of live CP after 7 days in culture; extensive staining is indicative cell viability. **(C)** Propidium iodide staining of live CP after 7 days in culture to detect dead cells; only very limited staining is observed. **(D,D')** CP organotypic culture treated with BrdU for 3 days and double-stained for BrdU and doublecortin (Dcx). Some double-labeled cells are observed. **(E)** Detection of NF200 and BrdU in an organotypic culture. Scale bars: **(A–C)** = 200 μm (**B** and **C** are at the same magnification); **(D,E)** = 50 μm.

Although expression of neural progenitor markers within the CP suggested that the Dcx-positive cells are born within the CP, it could not be ruled out that post-mitotic Dcx-positive neuroblasts had migrated into the CP from other brain regions. *In vivo* experiments cannot address this issue as BrdU would label both neural precursor cells born and matured in the CP and neuroblasts originating from adjacent brain regions that had migrated into the CP. Hence to address this issue, we set up CP organotypic cultures from E12 chick embryo that allow one to specifically study events occurring within the CP. As indicated by MTT metabolic assay, PI (Figures 7A–C) and Otx2 (not shown), the E12 organotypic CP cultures showed very good viability and maintenance of the CP phenotype over 7 days in culture. After 3 days labeling with BrdU, the CPs were stained either for BrdU and Dcx or BrdU and NF200 (Figures 7D–E). BrdU-positive cells were observed in the CP stroma, and some were found to be Dcx-positive; also a few BrdU and NF200 positive cells were observed. This suggests that the neuroblasts had been born within the CP.

## Discussion

This study shows that neurons are present from early stages of CP development and that they appear to originate from progenitor cells within the CP. Furthermore, it challenges the view that the CPs become innervated only postnatally, by clearly demonstrating innervation of the CP at early developmental stages.

### Proteins normally associated with neural progenitors are expressed in the immature developing CP

We have shown that the developing chick CPs express markers that are found in the neuroepithelium and/or radial glial cells of the developing central nervous system. Markers expressed in the neuroepithelium at early stages of development, including Sox2, vimentin, and ASPP2 (Bennett and Dilullo, 1985; Uwanogho et al., 1995; Sottocornola et al., 2010), have been found to be expressed in the epithelium of chick CPs. Expression of Sox2 in the prenatal mouse CP epithelium as well as in a subset of CP epithelial cells in the adult mouse has been previously reported (Ferri et al., 2004). This, together with our observations in chick and human CPs, indicates that expression of Sox2 in the CP is a feature conserved across species. Interestingly, whereas Sox2 staining intensity in both chick and human embryonic CPs is overall lower than in the neuroepithelium, some strongly Sox2-positive cells, mainly localized at CP branching points, are present. Differences in Sox2 levels have also been observed in the developing mouse telencephalon, with “high Sox2” radial glial cells displaying higher neurosphere forming ability, growth rate, and self-renewal capability than “low Sox2” intermediate progenitor cells (Hutton and Pevny, 2011). To establish whether the “high-Sox2” cells within the CPs are landmarks important for CP branching morphogenesis, and/or represent a neural stem cells population from which nestin and/or GFAP-positive cells in the stroma originate is an important and challenging question that will require extensive investigation.

In contrast to Sox2 and vimentin, the chick nestin-like protein, transitin, expressed in early radial glial cells that have neuronal differentiation potential (Kriegstein and Alvarez-Buylla, 2009), was not detected in the chick CP epithelium, but only in the mesenchyme. This was the case also for nestin in mouse and human CPs, where the nestin positive cells were located just beneath the CP epithelium as reported in neonatal rats (Huang et al., 2011). Cells expressing GFAP, another protein expressed in neural stem cells as well as in astrocytes, were found in the mesenchymal compartment of the chick CPs. Because of antibody species of origin, we could not double stain the chick CP for transitin and GFAP. However, double-staining of mouse E12.5 CPs for nestin and GFAP indicated that at least in this species the developing CP contains two distinct populations of “neural” cells in the stroma, identified by nestin and GFAP reactivity. Consistent with population(s) of neural progenitors within the CP was the presence of cells expressing the neuroblast marker, Dcx. The finding that BrdU incorporation in Dcx-positive cells was observed in isolated CPs, supports the view that the neurons present in the CP are born within the CP itself and originate from neural progenitors, rather than having migrated from the neighboring neuroepithelium. This is consistent with previous studies suggesting that cells isolated from the CP have neural potential, as they could form neurosphere-like structures and differentiated into neuronal-like cells (Itokazu et al., 2006).

**Figure 8:**
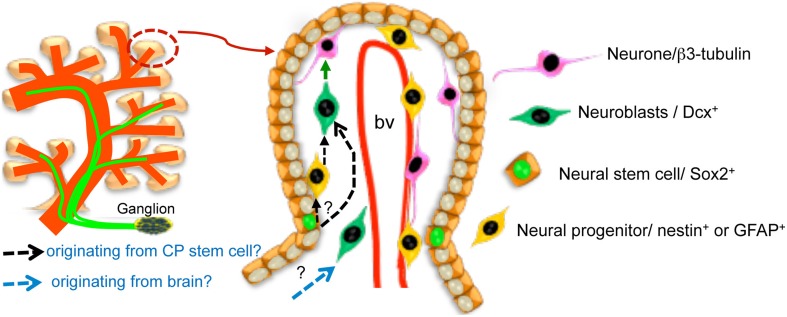
**Schematic representation of the CP neural components (innervation from ganglia outside the CP and neurons within the CP) and of the model proposing the existence of a neurovascular niche within the developing chick CP**. It is not currently known whether nestin-like-positive cells reflect a transition from high-Sox2/vimentin-positive cells to neuroblasts (black dotted arrows) nor whether some of the CP neurons are born from neuroblasts migrating into the CP from the brain rather than having been generated within it (blue dotted arrow). Please note that only the subset of highly Sox2-positive putative progenitors in the CP epithelium is indicated in the cartoon. The two sources are not mutually exclusive. bv, blood vessel.

The presence of subsets of highly Sox2-positive cells in the CP epithelium, that is also vimentin-positive, and of nestin-positive cells beneath it, raises the possibility, summarized in Figure 8, that the CP may be akin to the neurovascular niche in the subventricular region, where the neural stem cells reside close to the ependyma, in contact with blood vessels and extend their primary cilia into the CSF of the brain ventricles (Doetsch et al., 1997).

### Neurogenesis occurs within the developing CP

We have demonstrated for the first time that single neurons reside within the chick CP stroma from early stages of development. We have also shown that at least some of the neurons present in the developing CPs are catecholaminergic as indicated by co-expression of tyrosine-hydroxylase and β3-tubulin. This is consistent with the presence of dopamine receptors in the epithelium and smooth muscle cells of the blood vessels of the adult rat CP (Mignini et al., 2000).

Because of the difficulties in carrying out accurate quantification on neurons in whole mounts and variability among animals, we cannot be sure when neurons stop being born in the CP. Analysis of neurons in several preparations, and the fact that Dcx staining was more widespread around E12, would suggest that most neurons are born around this stage of development. However, some neurogenesis appeared to occur also at later stages, as some Dcx-BrdU labeled cells were observed after *in vivo* BrdU labeling at E15 (not shown).

The morphology of the CP neurons appears to change with development and the complexity of their branching appears to increase with embryonic age. CSF secretion is crucial for brain development and its production is at least in part controlled by the nervous system (Edvinsson and Lindvall, 1978; Lindvall and Owman, 1981). Projection into the CSF of fibers from individual neurons could be detected in some of the chick whole mount preparations, and fibers in very close proximity to the CP epithelium have been detected by light and transmission electron microscopy in adult CPs (Clark, 1928; Edvinsson et al., 1975; Nakamura and Milhorat, 1978). It is conceivable that these neurons act as sensory or autonomic neurons that integrate signals between the CP/CSF and the nerves entering the CP. The presence of numerous CSF-contacting axons has been reported in the ependyma and periventricular organs (Tramu et al., 1983; Michaloudi and Papadopoulos, 1996) and a mechanism for non-synaptic signal transmission in the brain has been proposed (Vigh et al., 2004).

### Innervation of the CP occurs pre-natally

The presence of nerves in adult CPs has been described in various species including mice, rats, hedgehogs, guinea-pigs, rabbits, cats, cows, and monkeys; differences in density of innervation were reported in different CPs, with the 3rd ventricle CP being the most densely innervated, and across species, with the lowest density of innervation observed in the mouse CP (Edvinsson et al., 1973; Lindvall et al., 1977a,b, 1978c; Lindvall, 1979). Notwithstanding its complexity, innervation of the CP was believed to occur postnatally (Tsuker, 1947; Lindvall and Owman, 1978). Our data clearly demonstrate that in the chick the nerves enter the CP before birth, apparently as early as at E8. The previously reported lack of evidence for prenatal innervation of the CP is likely due to the fact that it is very difficult to detect nerves, as well as neurons, in CP sections; apart from very early stages of development, clear visualization of CP innervation can be achieved only by using whole mount CP preparations and confocal microscopy; furthermore, nerves are easily pulled out of the CP during dissection, hence this step requires very careful handling. Expression of synaptic vesicle proteins involved in the release of neurotransmitters is indicative of neural activity within the CP well before birth, consistent with a role for innervation in the developing CP. Hence, innervation of the CP during development is in line with that of other secretory structures, such as the salivary glands that become innervated by parasympathetic nerves during organogenesis (Knox et al., 2010). As intestinal epithelial stem/progenitor cells in the crypts are controlled by mucosal afferent nerves (Lundgren et al., 2011), it is conceivable that the nerves might play a role in proliferation and branching of the CP. However, whether during development the nerves' main role is in controlling CSF secretion or also in CP growth remains to be established.

The nerves in the E20 CP did not appear to be myelinated, as indicated by lack of staining for myelin basic protein, and this is consistent with a transmission electron microscopy study of adult rat CP (Edvinsson et al., 1975; Nakamura and Milhorat, 1978).

The nature of CP innervation has been examined in a variety of species including adult rat, guinea-pig, hedgehog, rabbit, cat, and man; noradrenergic and cholinergic innervation, as well as substance P-positive fibers, have been described in adult CPs from different species, whereas the presence of serotoninergic fibers has so far been reported only in the rat and hedgehog CPs (Napoleone et al., 1982; Edvinsson et al., 1983; Nilsson et al., 1990; Michaloudi and Papadopoulos, 1996). However, most of these studies have not investigated comprehensively the presence of different types of fibers and their localization within the same CP. Hence, it will be important to carry out systematic comparative studies on the type of fibers present in developing and adult CP and innervation using whole mount preparations.

Another important finding that has emerged from staining whole mount preparations for β3-tubulin concerns the identification of a neural network between pineal gland, 3rd ventricle CP and lateral ventricle CP. This neural network may be important for development and maintenance of the CP throughout life as suggested in other organs, such as the developing teeth (Luukko et al., 1997), kidney (Tiniakos et al., 2004), pancreas (Burris and Hebrok, 2007), and heart (Hildreth et al., 2009). It is also tempting to speculate that in the developing chick, the neuron cluster between the 3rd and lateral ventricle CPs may serve as a relay station for integrating signals from the CSF and co-ordinating secretory activity throughout the ventricular system.

Together, as schematically summarized in Figure 8, the presence of innervation and of neurons adjacent to blood vessels, or projecting into the ventricles, is consistent with the hypothesis that the ventricular system contains a neural network that resembles to some extent the enteric nervous system and is important for CP development and homeostasis.

## Author contributions

WP designed and performed experiments, analyzed data and wrote the manuscript; BV and ZA performed experiments and critical reading of the manuscript; NJ performed experiments; PF planned research, analyzed data, and wrote the manuscript.

### Conflict of interest statement

The authors declare that the research was conducted in the absence of any commercial or financial relationships that could be construed as a potential conflict of interest.

## Supplementary material

Movie 1 **Neuron detected by ß3tubulin staining (red) in an E12 whole mount chick CP**. Nuclei are counterstained with Hoechst dye (blue). Scale bar = 100 μm.

Movie 2 **Neuron detected by ß3tubulin staining (red) coursing across the CP epithelium stained by the tight junction protein, ZO-1 (green), in an E12 whole mount chick CP**. Nuclei are counterstained with Hoechst dye (blue). Scale bar = 20 μm.

## Abbreviations

Aq-1: aquaporin-1
ASPP2: apoptosis-stimulating of p53 protein 2
BrdU: 5-bromo-2′-deoxyuridine
bv: blood vessel
CP: choroid plexus
CSF: cerebrospinal fluid
Dcx: doublecortin
EBSS: Earle's balanced salt solution
GAPDH: glyceraldehyde 3-phosphate dehydrogenase
GFAP: glial fibrillary acidic protein
HDBR: human developmental biology resource
ne: neuroepithelium
NF200: neurofilament 200 kDa
MB: midbrain
MBP: myelin basic protein
MTT: 3-(4,5-Dimethylthiazol-2-yl)-2,5-diphenyltetrazolium bromide
Otx2: orthodenticle homeobox 2
PFA: paraformaldehyde
pH3: phosphorylated-histone 3
PI: propidium iodide
RT-PCR: reverse transcription polymerase chain reaction
RT-qPCR: quantitative reverse transcription polymerase chain reaction
Sox2: SRY (sex determining region Y)-box 2
SV2: synaptic vesicles 2
TH: tyrosine-hydroxylase
vWF: von Willebrand factor
ZO-1: zonula occludens-1.
