# Supplementary figures and images for: The neural milieu of the developing choroid plexus: neural stem cells, neurons and innervation
Source: Front Neurosci. 2015 Mar 31;9:103. doi: 10.3389/fnins.2015.00103 (PMC4379892; doi:10.3389/fnins.2015.00103)

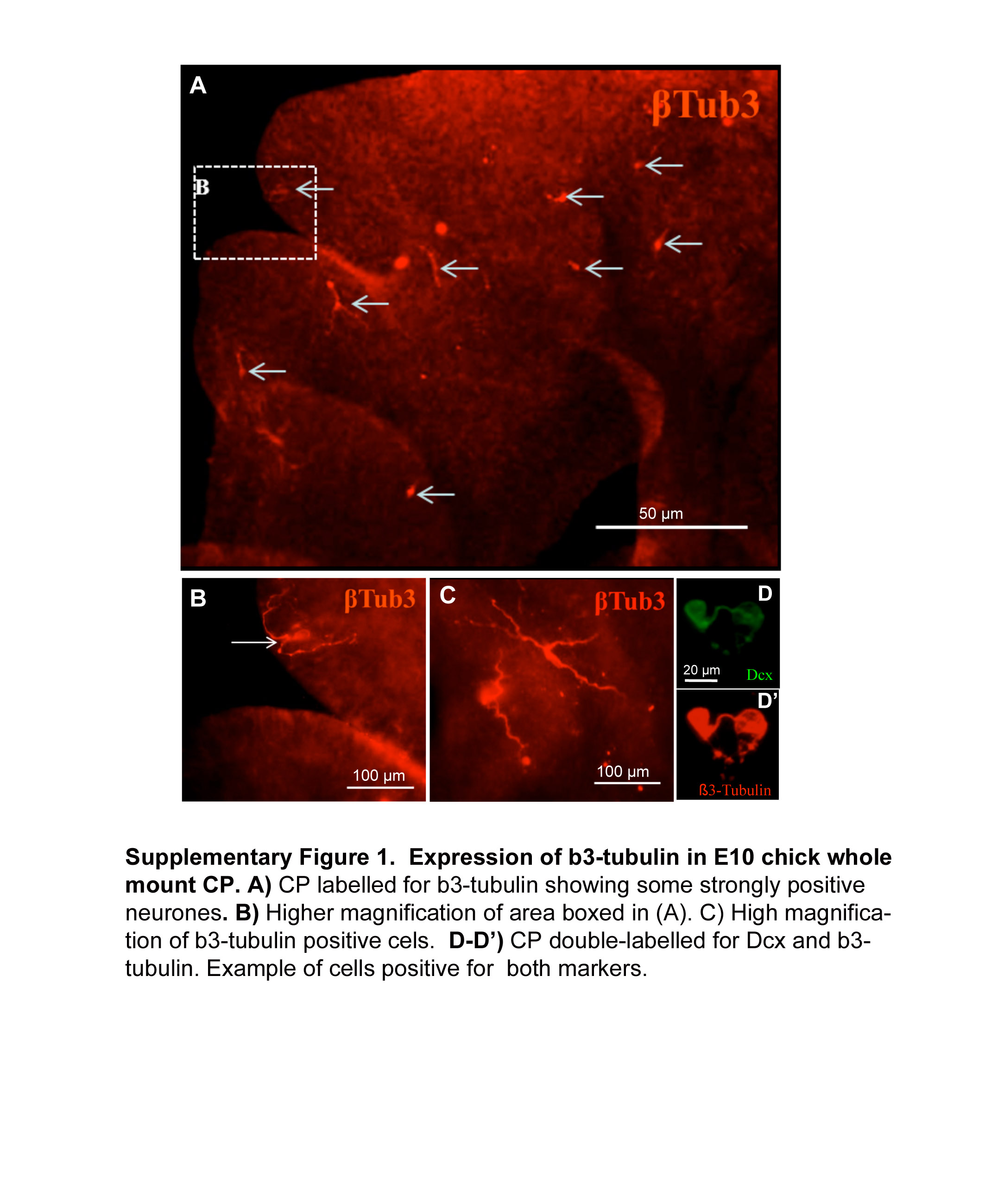

Supplement: Supplementary file 3 [file Image1.JPEG]

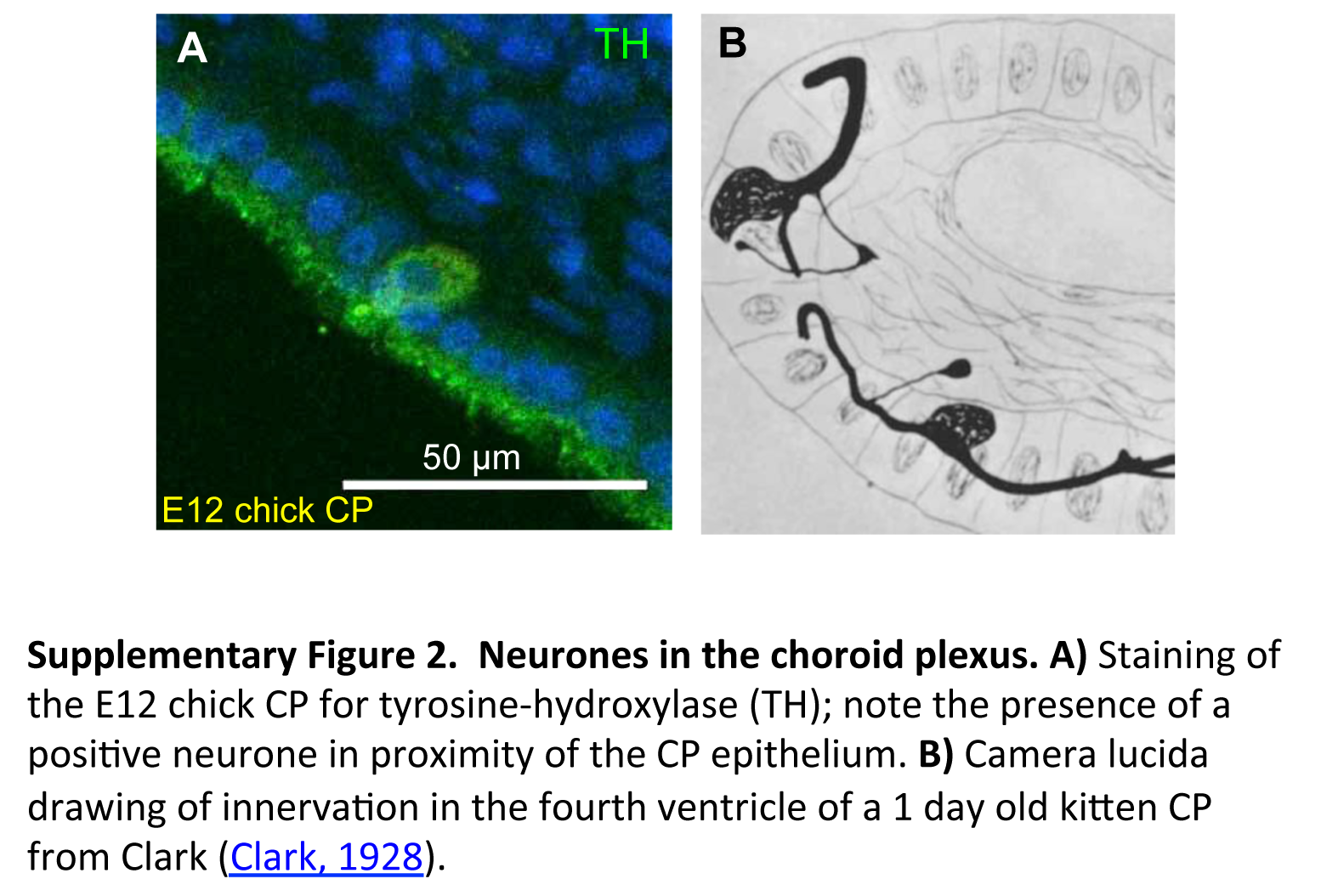

Supplement: Supplementary file 4 [file Image2.TIF]

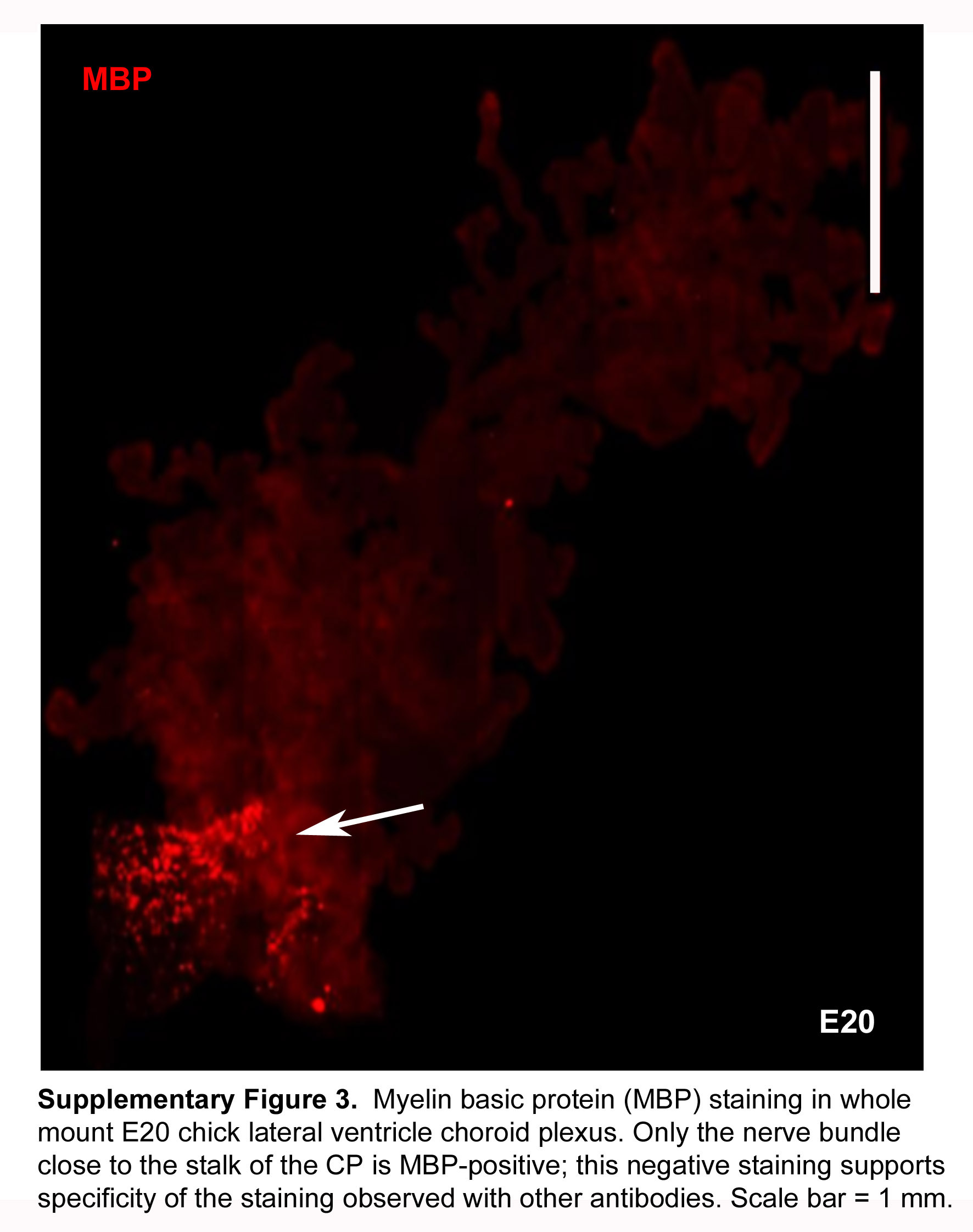

Supplement: Supplementary file 5 [file Image3.JPEG]
